# Thoracic and vertebral deformities in lung transplantation: perioperative complications and long-term prognoses

**DOI:** 10.1186/s12890-024-03168-6

**Published:** 2024-07-18

**Authors:** Etsuhiro Nikkuni, Takashi Hirama, Masahiro Ui, Toshikazu Watanabe, Shunta Mukai, Tatsuaki Watanabe, Yui Watanabe, Hisashi Oishi, Satoru Ebihara, Yoshinori Okada

**Affiliations:** 1 Department of Rehabilitation, Tohoku University Hospital, Sendai, Miyagi Japan; 2 Department of Thoracic Surgery, Institute of Development, Aging and Cancer, Tohoku University, Sendai, Miyagi Japan; 3 Division of Organ Transplantation, Tohoku University Hospital, Sendai, Miyagi Japan; 4 Department of Respiratory Medicine and Infectious Diseases, Niigata University Graduate School of Medical and Dental Sciences, Niigata, Niigata Japan; 5 Department of Respiratory Medicine, Fujita Health University School of Medicine, Toyoake, Aichi Japan; 6 Department of Rehabilitation Medicine, Tohoku University Graduate School of Medicine, Sendai, Miyagi Japan

**Keywords:** Lung transplant, Thoracic deformity, Vertebral deformity, Pectus excavatum, Scoliosis, Kyphosis

## Abstract

**Background:**

Lung transplantation (LTx) is a crucial therapeutic strategy for patients suffering from end-stage respiratory diseases, necessitating precise donor-recipient size matching to ensure optimal graft function. While standard allocation protocols rely on predicted lung capacity based on factors such as sex, age, and height, a subset of patients with respiratory diseases presents an additional challenge – thoracic or vertebral deformities. These deformities can complicate accurate volume predictions and may impact the success of lung transplantation.

**Methods:**

In this retrospective cohort study of patients who underwent LTx at Tohoku University Hospital between January 2007 and April 2022, with follow-up until October 2022, the primary objective was to assess the influence of thoracic and vertebral deformities on perioperative complications, emphasizing interventions, such as volume reduction surgery. The secondary objective aimed to identify any noticeable impact on long-term prognoses in recipients with these deformities.

**Results:**

Of 129 LTx recipients analyzed, 17.8% exhibited thoracic deformities, characterized by pectus excavatum, while 16.3% had vertebral deformities. Perioperative complications, requiring delayed chest closure, tracheostomy, and volume reduction surgery, were more prevalent in the deformity group. Thoracic deformities were notably associated with the need for volume reduction surgery. However, long-term prognoses did not differ significantly between patients with deformities and those without. Vertebral deformities did not appear to significantly impact perioperative or long-term outcomes.

**Conclusions:**

This study highlights the prevalence of thoracic deformities in LTx recipients, correlating with increased perioperative complications, particularly the potential need for volume reduction surgery. Importantly, these deformities do not exert a significant impact on long-term prognoses. Additionally, patients with vertebral deformities, such as scoliosis and kyphosis, appear to be manageable in the context of LTx.

**Supplementary Information:**

The online version contains supplementary material available at 10.1186/s12890-024-03168-6.

## Introduction

Lung transplantation (LTx) is a vital therapeutic approach for patients with end-stage respiratory diseases, aiming to extend the patients’ survival and enhance their quality of life. Donor-recipient size matching is a critical aspect of LTx surgery, ensuring optimal graft function [1–3]. During organ allocation, size matching is typically based on predicted total lung capacity (TLC) or predicted vital capacity (VC), which depend on such factors as sex, age, and height [4–6]. However, patients with respiratory diseases, particularly those with thoracic deformities, pose challenges in accurate volume predictions based solely on height. In such cases, there has been insufficient analysis of postoperative complications and long-term outcomes related to LTx. Theoretically, patients with thoracic deformities may encounter complexities arising from substantial lung deformities, elevating the risk of an oversized graft allocated according to predicted lung capacity. Such instances are anticipated to complicate perioperative management, necessitating interventions such as volume reduction surgery or delayed chest closure. Conversely, vertebral deformities may not exert a direct impact on lung volume reduction. However, the potential complications for patients with such deformities following LTx remain insufficiently documented. Consequently, the primary objective of this study is to retrospectively compare patients with pre-LTx thoracic deformities to those without, exploring the incidence of perioperative complications. The secondary objective involves assessing long-term prognoses in these distinct patient cohorts. The third objective is to retrospectively compare patients with pre-LTx vertebral deformities to those without, scrutinizing the incidence of perioperative complications and evaluating long-term prognoses in these patient cohorts.

## Materials and methods

### Study design and data collection

Patients who had undergone LTx at Tohoku University Hospital (TUH) from January 2007 to April 2022 were systematically enrolled as part of a retrospective cohort study, with follow-up observations extending through October 2022. The research cohort excluded individuals under the age of 18 and those who did not exhibit measurable thoracic or vertebral deformities. At the time of transplantation, fundamental data, denoted as pre-transplant data, were meticulously gathered. Postoperative follow-up data were collected monthly until discharge, and then every 6 months and annually thereafter.

### Ethics approval and consent to participate

It is imperative to emphasize that all research methodologies were executed in strict accordance with the principles of the Declaration of Helsinki. The study protocol received ethical approval from the Ethics Committee of Tohoku University Graduate School of Medicine, the requisite of obtaining informed consent having been waived in view of the retrospective nature of the study, which was assigned Institutional Review Board number 2021-1-866.

### Definition of thoracic deformity

**Fig. 1:**
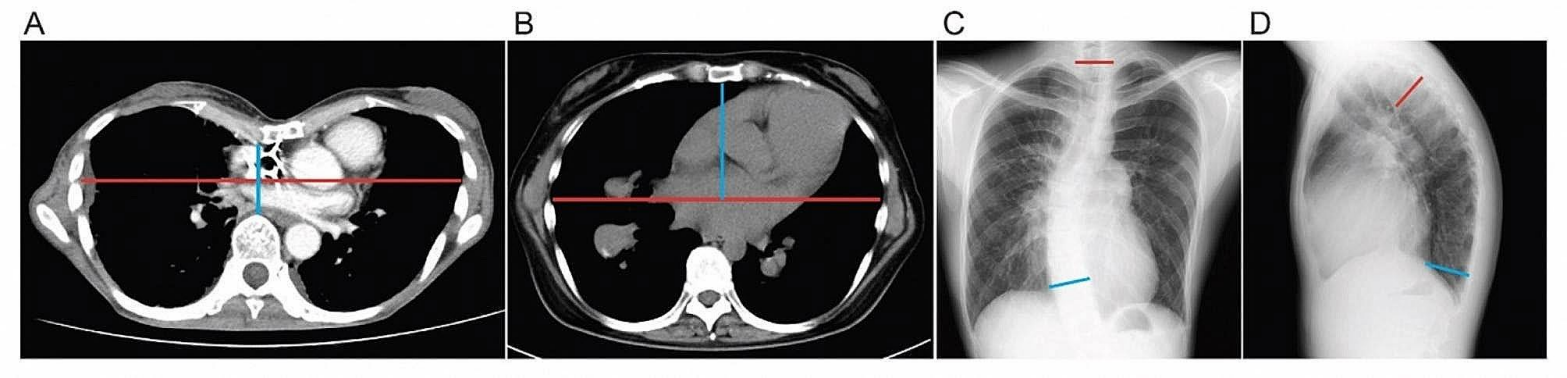
Measurement of thoracic and vertebral deformities and representative images. (**A**) Pectus excavatum was assessed using an axial chest CT image, and the Haller index was calculated by dividing the widest transverse diameter (red line) by the highest antero-posterior distance (blue line). (**B**) Pectus carinatum was assessed using an axial chest CT image, and the modified pectus index was calculated by dividing the widest transverse diameter (red line) by the distance from the central chord to the underside of the maximal protrusion (blue line). (**C**) Scoliosis was assessed using a posteroanterior chest radiograph, and the Cobb angle was calculated between the second and 11th thoracic vertebrae (shown by the red and blue lines, respectively). (**D**) Kyphosis was assessed using a lateral chest radiograph, and the Cobb angle was calculated between the fourth and 12th thoracic vertebrae (shown by the red and blue lines, respectively)

Thoracic deformity was defined by the presence of pectus excavatum or pectus carinatum (Table 1). Pectus excavatum was identified visually by a sunken appearance of the sternum and diagnosed by determining the Haller index by means of thoracic computed tomography (CT) [7–9]. This index represents the ratio between the widest transverse diameter and the antero-posterior distance from the sternum to the anterior surface of the vertebral body (Fig. 1). Pectus excavatum was diagnosed if the Haller index exceeded 3.2. In contrast, pectus carinatum was identified visually by a protrusion of the sternum and ribs and diagnosed by determining the modified pectus index by means of thoracic CT. This index represents the widest transverse diameter divided by the distance from the central chord to the undersurface of the maximal protrusion [10]. Pectus carinatum was diagnosed if the modified pectus index was less than 1.66.

**Table 1:** Definitions of vertebral and thoracic deformities

Type of Deformity	Image Selection	Measurement	Definition
Pectus excavatum	Axial view in chest CT scan	Haller index	> 3.2
Pectus carinatum	Axial view in chest CT scan	Modified pectus index	< 1.66
Scoliosis	Posteroanterior view in chest radiograph	Cobb angle of T2 and T11	> 10°
Kyphosis	Lateral view in chest radiograph	Cobb angle of T4 and T12	> 50°
Kyphoscoliosis	Posteroanterior and lateral views in chest radiographs		Meets criteria of both scoliosis and kyphosis

Thoracic deformity was determined visually and by the presence of either pectus excavatum or pectus carinatum in chest CT scans. Vertebral deformity was identified by the presence of scoliosis, kyphosis, or kyphoscoliosis in chest radiographs

### Definition of vertebral deformity

Vertebral deformity was defined by the appearance of scoliosis, kyphosis, or kyphoscoliosis in chest radiographs taken at the time of transplantation (Table 1). Scoliosis was diagnosed if the Cobb angle formed between the upper part of the second thoracic vertebra and the lower part of the 11th thoracic vertebra in a posterior-anterior chest radiograph exceeded 10° [11, 12] (Fig. 1). Kyphosis was diagnosed if the Cobb angle between the upper part of the fourth thoracic vertebra and the lower part of the 12th thoracic vertebra in a lateral chest radiograph exceeded 50°. Kyphoscoliosis was diagnosed by the presence of both scoliosis (T2/T11 Cobb angle > 10°) and kyphosis (T4/T12 Cobb angle > 50°) [13]. Those with characteristics of both thoracic and vertebral deformities were termed overlap deformity.

### Management of LTx recipients

Our center has previously reported on various aspects of post-transplant management, including the administration of immunosuppressive drugs [14], prophylactic antibiotics during the perioperative and chronic phases [15, 16], histocompatibility testing [17], and general chronic disease management and rehabilitation [18, 19]. At our institution, bilateral LTx is conducted utilizing a clamshell incision, whereas single LTx is undertaken employing a unilateral anterolateral incision and transverse sternotomy or posterolateral thoracotomy according to patient’s condition and transplant side. Primary chest closure is considered the standard procedure; however, in consideration of hemodynamic stability, pulmonary edema, and/or graft size-matching, the responsible surgeon may opt for delayed chest closure. Volume reduction surgery is performed only in cases where chest closure is deemed challenging during primary chest closure or delayed chest closure.

### Variables in the study

Body weight was assessed using Body Mass Index (BMI) as the metric, categorized as Underweight (BMI < 18.5 kg/m2), Normal (BMI 18.5–24.9), and Overweight (BMI > 25.0) [20]. The criteria for diagnosing chronic lung allograft dysfunction (CLAD) was a sustained (≥ 3 months) and substantial (≥ 20%) reduction in FEV1 from the baseline value, extending beyond six months after transplantation [17, 19]. Use of intraoperative extracorporeal life support (ECLS), including off-pump, extracorporeal membrane oxygenation (ECMO), and cardiopulmonary bypass (CPB), was reviewed [21]. The definition and grading of primary graft dysfunction (PGD) were established according to the relevant guidelines from the International Society for Heart and Lung Transplantation. Grade 3 PGD involves the use of ECLS or mechanical ventilation with an inspired oxygen fraction > 0.5 on nitric oxide for more than 48 h post-lung transplantation [22, 23].

### Data analysis

The data regarding variables comparing vertebral and thoracic deformities with controls were presented as the number with percentages or medians with interquartile ranges (IQR), as appropriate. To assess differences in baseline data, categorical variables were evaluated using the chi-square test, while continuous variables were analyzed using the Kruskal-Wallis test. The identification of risk factors associated with volume reduction surgery was conducted through the use of multivariable logistic regression models. Clinically important variables such as age, gender, LTx indication, and thoracic deformity were selected, and the analysis was performed using forward-stepwise selection, choosing predictors deemed significant. Due to the limited sample size, the analysis was restricted to two predictor variables. Time-to-event outcomes were modeled using the Kaplan-Meier method, and group differences were determined with the log-rank test. Statistical significance was defined as *p* values < 0.05. Statistical analyses and the creation of graphs were performed using GraphPad Prism 10 (GraphPad Software, Inc., La Jolla, CA).

## Results

### Prevalence of thoracic and vertebral deformities in LTx recipients

Of the 139 patients who underwent LTx between 2007 and 2022, two were excluded because of the inability to assess thoracic or vertebral deformities, and an additional eight pediatric cases were also omitted from the analysis. Consequently, the study encompassed a total of 129 LTx recipients. The determination of their thoracic or vertebral deformity status was established through evaluations conducted using chest radiographs and thoracic CT scans, all conducted prior to the LTx procedure. Among the 129 LTx recipients, 23 (17.8%) were classified as having thoracic deformities, all of whom exhibited pectus excavatum, with no cases demonstrating pectus carinatum. Additionally, within this cohort, 21 patients (16.3%) were identified as having vertebral deformities, comprising ten cases of scoliosis, nine of kyphosis, and two of kyphoscoliosis. Cases with overlap deformity were categorized separately, resulting in the analysis of four groups: thoracic deformity (*n* = 18), vertebral deformity (*n* = 16), overlap deformity (*n* = 5), and non-deformity (control, *n* = 90), as summarized in Table 2.

**Table 2:** Clinical characteristics of LTx recipients with thoracic and vertebral deformities

	Total *n* = 129	Control *n* = 90	Thoracic Deformity *n* = 18	Vertebral Deformity *n* = 16	Overlap Deformities *n* = 5	*P*-value
Type of deformity, n (%)						
*- Pectus excavatum*	23 (17.8%)		18 (100%)		5 (100%)	
*- Pectus carinatum*	0 (0%)		0 (0%)		0 (0%)	
*- Scoliosis*	10 (7.8%)			6 (37.5%)	4 (80.0%)	
*- Kyphosis*	9 (7.0%)			9 (56.3%)	0 (0%)	
*- Kyphoscoliosis*	2 (1.6%)			1 (6.3%)	1 (20.0%)	
Age at LTx, median (IQR)	45 (35–52)	46 (34–51)	38 (30–48)	51 (42–56)	43 (34–53)	0.158
Sex, female, n (%)	77 (59.7%)	53 (58.9%)	14 (77.8%)	7 (43.8%)	3 (60.0%)	0.245
Body-mass index (kg/m^2^), n (%)						0.053
*- <18.5: underweight*	67 (51.9%)	39 (43.3%)	15 (83.3%)	9 (56.3%)	4 (80.0%)	
*- 18.5–24.9: normal*	48 (37.2%)	38 (42.2%)	3 (16.7%)	6 (37.5%)	1 (20.0%)	
*- ≥25.0: overweight*	14 (10.9%)	13 (14.4%)	0 (0%)	1 (6.3%)	0 (0%)	
LTx procedure, n (%)						0.628
*- Single*	76 (58.9%)	55 (61.1%)	7 (38.9%)	11 (68.8%)	3 (60.0%)	
*- Bilateral*	45 (34.9%)	30 (33.3%)	9 (50.0%)	4 (25.0%)	2 (40.0%)	
*- Living-donor*	8 (6.2%)	5 (5.6%)	2 (11.1%)	1 (6.3%)	0 (0%)	
LTx indication, n (%)						0.011
*- Obstructive*	49 (38.0%)	39 (43.3%)	2 (11.1%)	8 (50.0%)	0 (0%)	
*- Vascular*	24 (18.6%)	19 (21.1%)	3 (16.7%)	1 (6.3%)	1 (20.0%)	
*- Suppurative*	13 (10.1%)	8 (8.9%)	2 (11.1%)	3 (18.8%)	0 (0%)	
*- Fibrosis*	35 (27.1%)	21 (23.3%)	9 (50.0%)	3 (18.8%)	2 (40.0%)	
*- Allogeneic*	8 (6.2%)	3 (3.3%)	2 (11.1%)	1 (6.3%)	2 (40.0%)	
Donor age, median (IQR)	44 (35–53)	44 (35–53)	53 (48–61)	42 (28–57)	43 (35–44)	0.033
Donor age, > 55, n (%)	23 (18.5%)	11 (12.6%)	7 (41.2%)	5 (33.3%)	0 (0%)	0.138
Ratio of predicted VC R/D (%), median (IQR)	95.8 (86.6–104.5)	104.1 (95.4-114.6)	98.0 (91.5-111.8)	113 (98.0-119.3)	104.0 (102.0-115.0)	0.488
Ischemic time (min), median (IQR)	494 (431–652)	494 (438–654)	480 (360–688)	498 (392–637)	483 (402–544)	0.725
Operation time (min), median (IQR)	494 (393–805)	492 (389–818)	620 (473–936)	432 (367–769)	528 (470–708)	0.396
Intraoperative ECLS use, n (%)						0.075
*- CPB*	34 (26.4%)	25 (27.8%)	6 (33.3%)	2 (12.5%)	1 (20.0%)	
*- ECMO*	59 (45.7%)	41 (45.6%)	11 (61.1%)	5 (31.3%)	2 (40.0%)	
*- Off-pump*	36 (27.9%)	24 (26.7%)	1 (5.6%)	9 (56.3%)	2 (40.0%)	
Delayed chest closure, n (%)	33 (25.6%)	17 (18.9%)	11 (61.1%)	2 (12.5%)	3 (60.0%)	< 0.001
Volume reduction, n (%)	22 (17.1%)	11 (12.2%)	5 (27.8%)	3 (18.8%)	3 (60.0%)	0.023
Tracheostomy, n (%)	61 (47.3%)	41 (45.6%)	13 (72.2%)	4 (25.0%)	3 (60.0%)	0.044
Invasive mechanical ventilation (day), median (IQR)	10 (3–29)	8 (3–26)	21 (13–37)	5 (2–18)	21 (6–40)	0.015
ICU stay (day), median (IQR)	18 (9–35)	16 (8–35)	31 (19–39)	11 (6–26)	36 (12–46)	0.034
PGD†						0.601
*- grade 0*	24 (19.4%)	16 (18.4%)	2 (11.8%)	5 (33.3%)	1 (20.0%)	
*- grade 1*	28 (22.6%)	20 (23.0%)	4 (23.5%)	3 (20.0%)	1 (20.0%)	
*- grade 2*	27 (21.8%)	19 (21.8%)	2 (11.8%)	5 (33.3%)	1 (20.0%)	
*- grade 3*	45 (36.3%)	32 (36.8%)	9 (52.9%)	2 (13.3%)	2 (40.0%)	
Three-month mortality, n (%)	8 (6.2%)	4 (4.4%)	3 (22.2%)	1 (6.3%)	0 (0%)	0.241
Follow-up period (month), median (IQR)	62 (28–109)	65 (37–110)	45 (5-115)	43 (17–90)	59 (29–128)	0.398

LTx: lung transplant; IQR: interquartile range; VC: vital capacity; R: recipient; D: donor; ECLS: extracorporeal life support; CPB: cardiopulmonary bypass; ECMO: extracorporeal membrane oxygenation; ICU: intensive care unit; PGD: primary graft dysfunction

† missing data of 3 in control, 1 in thoracic and 1 in vertebral deformities

### Early outcomes in LTx recipients with thoracic deformity

In this study, 129 patients were classified into four groups based on the type of deformity, with 18 categorized as isolated thoracic deformity and 5 as the overlap group. There were no significant differences in recipient age or female predominance between patients with thoracic deformities and other groups. However, individuals with thoracic deformities tended to have a leaner physique before transplantation compared to those in other groups, with no overweight individuals observed in this group (Table 2). The indications for LTx varied significantly: cases with thoracic and overlap deformities had a lower prevalence of obstructive disorders (11.1% and 0%, respectively) and a higher occurrence of fibrotic disorders (50% and 40%, respectively) (*p* = 0.011). Moreover, the median donor age was significantly higher in the thoracic deformity group at 53 years (IQR 48–61) compared to the control group and vertebral deformity group, which had median ages of 44 years (IQR 35–53) and 42 years (IQR 28–57), respectively (*p* = 0.033). Intraoperative conditions, including ischemic time, operation duration, and ECLS use, appeared similar among the groups. However, the post-operative course was more complex in the thoracic deformity cohort. Patients with thoracic and overlap deformities underwent more procedures, such as delayed chest closure (61.1% and 60%, respectively, compared to 18.9% in the control group) and tracheostomy (72.2% and 60%, compared to 45.6% in the control group). Additionally, they required longer periods of mechanical ventilation, with a median duration of 21 days (IQR 13–37) and intensive care, with a median duration of 31 days (IQR 19–39). In contrast, the control group or vertebral deformity group required mechanical ventilation for a shorter median duration of 8 days (IQR 3–26) and 5 days (IQR 2–18), respectively, and intensive care for a shorter median duration of 16 days (IQR 8–35) and 11 days (IQR 6–26), respectively. Grade 3 PGD tended to be higher in the thoracic deformity group (52.9%) compared to the control group (36.8%) or vertebral deformity group (13.3%) without statistical significance.

While the ratio of predicted VC in the donor/recipient remained consistent between the thoracic deformity group and the control group (98.0% vs. 104.1%, *p* = 0.488), it is worth highlighting that LTx recipients with thoracic and overlap deformities underwent more volume reduction surgery (27.8% and 60%, respectively) than those with vertebral deformity or without thoracic deformities (18.8% and 12.2%, *p* = 0.023) (Table 2). Among the 22 patients who underwent volume reduction surgery, 65.2% underwent wedge resection, 17.4% underwent lobectomy, and 13.0% underwent combined wedge resection and lobectomy. The resected locations, with duplications, included the right upper lobe (50.0%), right middle lobe (72.7%), right lower lobe (13.6%), upper segments of the left upper lobe (54.5%), lingular segments of the left upper lobe (77.3%), and left lower lobe (18.2%). In Japan, deceased-donor lung allocation primarily relies on the predicted VC of recipients, with minimal consideration given to chest-wall deformities. Consequently, this approach often results in situations where the donor’s lung volume exceeds the recipient’s chest cavity, especially in cases of thoracic deformity, leading to a higher likelihood of volume reduction surgery. An additional analysis of risk factors for volume reduction was conducted using multivariable logistic regression models (Table 3), suggesting a potential association between thoracic deformity and the need for volume reduction (odds ratio [OR] 3.79, 95% CI 1.28–11.11, *p* = 0.021). Similarly, sensitivity analysis with four variables indicated comparable results (OR 3.91, 95% CI 1.22–12.65, *p* = 0.21) as shown in the **Supplemental Table**.

**Table 3:** Risk factors associated with volume reduction

Variables	Odds Ratio	95%CI	*P*-value
Fibrosis	0.74	0.23–2.12	0.592
Thoracic deformity	3.79	1.28–11.11	0.021

CI: 95% confidence interval

### Long-term outcomes of LTx recipients with thoracic deformity

**Fig. 2:**
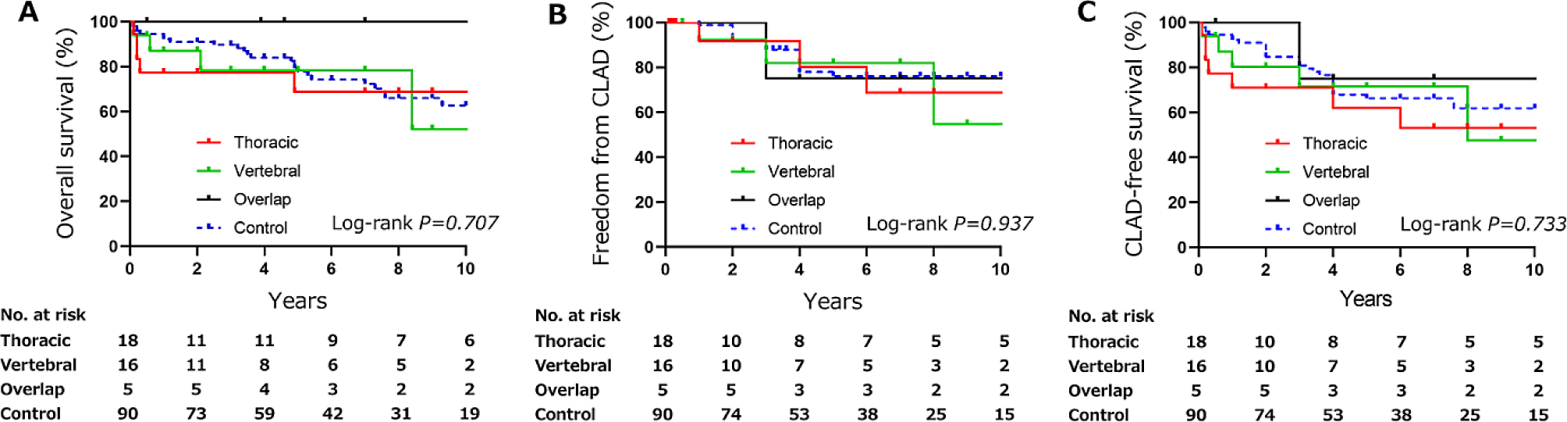
Long-term outcomes of LTx recipients with thoracic and vertebral deformities. (**A**) Overall survival was determined by considering death from any cause to be an event. (**B**) Freedom from chronic lung allograft dysfunction (CLAD) was defined as the occurrence of CLAD. (**C**) CLAD-free survival was determined on the basis of events that included both the development of CLAD and death from any cause. For cases without any events, the date of the last follow-up was used for censoring. The number of patients at risk was recorded at different time points

Despite the observed complex post-operative morbidities in patients with thoracic deformities, our analysis did not find statistical significance in the three-month mortality rates between these patients and those without thoracic deformities (Table 2). However, there was a numerical disparity in the three-month mortality rates (22.2% in thoracic deformity vs. 6.3% in vertebral deformity or 4.4% in control), suggesting a potential impact of thoracic deformities on survival outcomes. Therefore, we extended our investigative focus to long-term prognosis based on three pivotal parameters: overall survival, freedom from CLAD, and CLAD-free survival (Fig. 2). It is noteworthy that, over the course of our longitudinal follow-up, extending until October 2022 with median followup of 62 months (IQR 28–109), the presence of thoracic deformities exhibited no association with heightened mortality rates as compared to the absence of thoracic deformities (log-rank *p* = 0.707). Additionally, the incidence of CLAD within our cohort exhibited a comparable trend between the two groups (log-rank *p* = 0.937), and the trajectory of CLAD-free survival exhibited analogous patterns for patients both with and without thoracic deformities (log-rank *p* = 0.733).

### Early and long-term outcomes in LTx recipients with vertebral deformity

Among the 21 patients with vertebral deformities, 16 were categorized as having isolated vertebral deformity, while five were classified in the overlap group (Table 2). No statistically significant differences were observed in age, sex, body mass index, or type of transplant procedure (single, bilateral, and living-donor) at the time of LTx between patients with vertebral deformities and those in other groups. Additionally, there were no significant differences in the predicted VC ratio between donor and recipient, ischemic time, or intraoperative ECLS use between the vertebral deformity group and other groups. Regarding volume reduction procedures, no statistically significant differences were observed between the vertebral deformity group and the others. Post-operative morbidity and mortality were thoroughly assessed, revealing no substantial differences in terms of tracheostomy, duration of invasive mechanical ventilation, proportion of PGD grades, and three-month mortality among patients with vertebral deformities. In addition to examining short-term outcomes, we conducted a comprehensive assessment of long-term outcomes using the Kaplan-Meier model (Fig. 2). Our analysis, which included follow-up data until October 2022, showed that the presence of vertebral deformities before LTx did not indicate an increased risk of mortality compared to the other groups. Likewise, there were no significant differences observed in freedom from CLAD or CLAD-free survival between patients with and without vertebral deformities.

## Discussion

Thoracic and vertebral deformities are common clinical complications, yet there is a lack of comprehensive studies addressing their impact on lung transplantation. Therefore, we conducted a single-center analysis to investigate how these deformities affect lung transplantation outcomes, both in the short and long term. At our center, we found that 17.8% of lung transplant (LTx) recipients had thoracic deformities, while 16.3% had vertebral deformities. A representative study on the impact of thoracic deformities on prognosis was conducted by Sonaglioni et al., who reported a significantly reduced survival period in individuals with idiopathic pulmonary fibrosis (IPF) who also had chest-wall deformities, compared to those without such complications [24]. However, studies specifically focusing on lung transplantation are limited. The candidate selection consensus proposed by the International Society for Heart and Lung Transplantation highlights the presence of restrictive chest-wall or vertebral deformities as significant risk factors [25]. However, a study by Miyahara et al. examining five-year survival rates and the incidence of CLAD in 30 cases of lung transplantation involving chest-wall deformities and 38 cases without such complications found no significant differences in survival rates or CLAD incidence between the two groups [26]. When incorporating our analysis into these previous reports, it appears that thoracic deformities have a limited influence on the long-term prognoses of lung transplantation.

The long-term prognoses do not exhibit significant disparities between the cohort with thoracic deformities and the control group; nevertheless, a pronounced discrepancy becomes evident when assessing perioperative complications (Table 2). This discrepancy encompasses not only an elevated incidence of delayed chest closure and tracheostomy but also a notable escalation in the necessity for volume reduction surgery. Our analysis underscores that thoracic deformities represent a discernible risk factor necessitating volume reduction surgery (Table 3). It is worth noting that Montoya et al. have documented that patients undergoing graft reduction surgery in the context of LTx experience a decrement in respiratory function, resulting in reduced life expectancy [27]. Furthermore, Miyahara et al. have reported a notable predilection for LTx recipients with chest-wall deformities to undergo downsizing of the transplanted lung compared to their counterparts devoid of such deformities [26]. Consistent with recommendations made by Riddell et al., an optimal approach involves the use of grafts matched to the recipient’s anatomical dimensions [2]. Regrettably, thoracic deformities are not usually considered when organs are allocated, forcing transplant centers to resort to undersized grafts on such candidates when necessary. It is imperative to recognize that in countries with a severe donor shortage, such as Japan, the luxury of awaiting a perfectly matched graft may be infeasible, and decisions regarding graft acceptance must often be expedited, frequently during the initial donor call. Of particular interest, there are reports suggesting the feasibility of concurrently addressing corrective surgery for chest deformity and LTx, potentially rendering this approach a viable therapeutic option, particularly in cases of severe thoracic deformities [28]. This intriguing avenue warrants further investigation and consideration within the context of LTx.

Vertebral deformities such as scoliosis and kyphosis are considered contributing factors to reduced pulmonary function and weakened respiratory muscles [29]. Severe scoliosis can limit the mobility of the chest cage and spine [30]. While there is no systematic review on how vertebral deformities impact prognoses of lung transplantation, numerous case reports exist. Yamamoto et al. reported the case of a 14-year-old female with interstitial pneumonia accompanied by scoliosis (Cobb angle of 65°) who underwent LTx developing bronchial stenosis postoperatively but maintaining a good outcome for over five years [31]. Su et al. reported two cases of LTx in patients with scoliosis [32]: the first was a 53-year-old female with sarcoidosis, presenting with a Cobb angle of 80°; the second, a 46-year-old female with Kartagener syndrome presenting with a Cobb angle of 72°. Despite both cases exhibiting severe scoliosis, the postoperative courses were favorable. Moreover, Fukahara et al. reported the case of a 17-year-old female with congenital heart disease and kyphoscoliosis who underwent spine fixation surgery, followed by heart-lung transplantation, with a good outcome six months postoperatively [33]. At our center, we also believe that vertebral distortions or deformities do not significantly impact the perioperative or long-term outcomes of LTx and thus do not constitute absolute technical contraindications but, rather, are factors that require careful consideration.

The limitations of this study include its single-center nature, retrospective design, and susceptibility to unaccounted-for confounding variables. Conducting the study at a single center may have led to findings that do not fully represent the broader diversity of practices and patient demographics across different transplant centers. The retrospective approach, relying on existing medical records, may introduce inherent biases and limitations in data collection. Establishing a clear cause-and-effect relationship poses challenges in retrospective studies. The temporal sequence of events, including post-operative complications or long-term prognoses, is less precise compared to prospective studies. Incorporating prospective studies could offer a more robust solution to these challenges. While efforts were made to control for certain factors, the study may not encompass all potential confounding variables, such as comorbidities, socioeconomic factors, and variations in surgical techniques, which could impact the observed outcomes. Specifically, the higher donor age in the thoracic deformity group, with older donors generally considered as extended donors [34], may influence the risk of perioperative complications, potentially affecting the study’s results. These limitations underscore the need for cautious interpretation of the findings and warrant consideration of further multi-center research to enhance the generalizability and depth of understanding in this area.

## Conclusions

Our study highlights the prevalence of thoracic deformities, including pectus excavatum, in LTx recipients, and their association with increased perioperative complications, potentially leading to the need for volume reduction surgery. Importantly, these deformities do not exert a significant impact on long-term prognoses. Additionally, patients with vertebral deformities, such as scoliosis and kyphosis, appear to be manageable in the context of LTx. Despite some limitations, these findings underscore the significance of adopting personalized approaches in organ allocation and surgical decision-making for LTx candidates with thoracic and vertebral deformities.

### Electronic supplementary material

Below is the link to the electronic supplementary material.

Supplementary Material 1

## Data Availability

The datasets used during the current study are available from the corresponding author on reasonable request.

## Abbreviations

CLAD: Chronic lung allograft dysfunction
D: Donor
ECLS: Extracorporeal life support
ICU: Intensive care unit
IQR: Interquartile range
LTx: Lung transplant
R: Recipient
VC: Vital capacity

## References

[1] Chambers DC, Cherikh WS, Harhay MO, Hayes D, Hsich E, Khush KK (2019). The international thoracic Organ Transplant Registry of the International Society for Heart and Lung Transplantation: thirty-sixth adult lung and heart-lung transplantation Report-2019; focus theme: Donor and recipient size match. J Heart Lung Transpl.

[2] Riddell P, Ma J, Dunne B, Binnie M, Cypel M, Donahoe L (2021). A simplified strategy for donor-recipient size-matching in lung transplant for interstitial lung disease. J Heart Lung Transpl.

[3] Mangukia C, Shigemura N, Stacey B, Sunagawa G, Muhammad N, Espinosa J (2021). Donor quality assessment and size match in lung transplantation. Indian J Thorac Cardiovasc Surg.

[4] Jin Z, Hana Z, Alam A, Rajalingam S, Abayalingam M, Wang Z (2020). Review 1: lung transplant-from donor selection to graft preparation. J Anesth.

[5] Ehrsam JP, Held U, Opitz I, Inci I (2020). A new lung donor score to predict short and long-term survival in lung transplantation. J Thorac Dis.

[6] Hirama T, Akiba M, Watanabe T, Watanabe Y, Notsuda H, Oishi H (2022). Waitlist Mortality in lung transplant candidates in Japan. Transplantation.

[7] Robbins LP (2011). Pectus Excavatum. Radiol Case Rep.

[8] Shaalan AM, Kasb I, Elwakeel EE, Elkamali YA (2017). Outcome of surgical repair of Pectus Excavatum in adults. J Cardiothorac Surg.

[9] Kim Y-J, Heo J-Y, Hong K-H, Lim IH, Lee B-Y (2018). Computer-aided design and Manufacturing Technology for Identification of Optimal Nuss Procedure and Fabrication of Patient-Specific Nuss Bar for minimally invasive surgery of Pectus Excavatum. Appl Sci.

[10] Martinez-Ferro M (2016). Indexes for Pectus deformities. Chest wall deformities and corrective procedures.

[11] Kuznia AL, Hernandez AK, Lee LU (2020). Adolescent idiopathic scoliosis: common questions and answers. Am Fam Physician.

[12] Horng M-H, Kuok C-P, Fu M-J, Lin C-J, Sun Y-N (2019). Cobb Angle Measurement of Spine from X-Ray images using convolutional neural network. Comput Math Methods Med.

[13] Koelé MC, Lems WF, Willems HC (2020). The clinical relevance of hyperkyphosis: a narrative review. Front Endocrinol (Lausanne).

[14] Katahira M, Hirama T, Eba S, Suzuki T, Notsuda H, Oishi H (2020). Impact of postoperative continuous renal replacement therapy in lung transplant recipients. Transpl Direct.

[15] Hirama T, Tomiyama F, Notsuda H, Watanabe T, Watanabe Y, Oishi H (2021). Outcome and prognostic factors after lung transplantation for bronchiectasis other than cystic fibrosis. BMC Pulm Med.

[16] Ui M, Hirama T, Akiba M, Honda M, Kikuchi T, Okada Y (2023). Cellular and humoral immune responses after a third dose of SARS-CoV-2 mRNA vaccine in lung transplant recipients in Japan. Vaccine.

[17] Kumata S, Hirama T, Watanabe Y, Oishi H, Niikawa H, Akiba M (2020). The fraction of sensitization among lung transplant recipients in a transplant center in Japan. BMC Pulm Med.

[18] Hirama T, Okada Y (2023). Roles of respirologists in lung transplantation in Japan: narrative review. J Thorac Dis.

[19] Nikkuni E, Hirama T, Hayasaka K, Kumata S, Kotan S, Watanabe Y (2021). Recovery of physical function in lung transplant recipients with Sarcopenia. BMC Pulm Med.

[20] Purnell JQ. Definitions, classification, and epidemiology of obesity. 2000. 25905390

[21] Ohsumi A, Date H (2021). Perioperative circulatory support for lung transplantation. Gen Thorac Cardiovasc Surg.

[22] Snell GI, Yusen RD, Weill D, Strueber M, Garrity E, Reed A (2017). Report of the ISHLT Working Group on Primary Lung Graft Dysfunction, part I: definition and grading—A 2016 Consensus Group statement of the International Society for Heart and Lung Transplantation. J Heart Lung Transplantation.

[23] Hirama T, Akiba M, Watanabe T, Watanabe Y, Oishi H, Okada Y (2024). A single-center analysis of how HLA Mismatch and Donor-Specific antibodies affect short-term Outcome after Lung Transplantation: a pilot study before a country-wide histocompatibility study in Japan. Transpl Proc.

[24] Sonaglioni A, Caminati A, Nicolosi GL, Lombardo M, Harari S (2022). Influence of chest wall conformation on spirometry parameters and outcome in mild-to-moderate idiopathic pulmonary fibrosis. Intern Emerg Med.

[25] Leard LE, Holm AM, Valapour M, Glanville AR, Attawar S, Aversa M (2021). Consensus document for the selection of lung transplant candidates: an update from the International Society for Heart and Lung Transplantation. J Heart Lung Transpl.

[26] Miyahara S, Chen-Yoshikawa TF, Motoyama H, Nakajima D, Hamaji M, Aoyama A (2019). Impact of flat chest on cadaveric lung transplantation: postoperative pulmonary function and survival. Eur J Cardiothorac Surg.

[27] Montoya P, Bello I, Ascanio F, Romero L, Pérez J, Rosado J (2021). Graft reduction surgery is associated with poorer outcome after lung transplantation: a single-centre propensity score-matched analysis. Eur J Cardiothorac Surg.

[28] Rahimi N, Matilla JR, Lang G, Schwarz S, Nachbaur E, Benazzo A (2021). Simultaneous pectus excavatum correction and lung transplantation-A case series. Am J Transpl.

[29] Koumbourlis AC (2006). Scoliosis and the respiratory system. Paediatr Respir Rev.

[30] Leong JC, Lu WW, Luk KD, Karlberg EM (1999). Kinematics of the chest cage and spine during breathing in healthy individuals and in patients with adolescent idiopathic scoliosis. Spine (Phila Pa 1976).

[31] Yamamoto H, Otani S, Miyoshi K, Sugimoto S, Yamane M, Toyooka S (2021). Long-term clinical follow-up after lung transplantation in patient with scoliosis: a case report. Gen Thorac Cardiovasc Surg.

[32] Su JW, Mason DP, Murthy SC, Budev MM, Mehta AC, Goodwin R (2008). Successful double lung transplantation in 2 patients with severe scoliosis. J Heart Lung Transpl.

[33] Fukahara K, Minami K, Hansky B, Schulte-Eistrup SA, Tenderich G, Schulz U (2003). Successful heart-lung transplantation in a patient with kyphoscoliosis. J Heart Lung Transpl.

[34] Pierre AF, Sekine Y, Hutcheon MA, Waddell TK, Keshavjee SH (2002). Marginal donor lungs: a reassessment. J Thorac Cardiovasc Surg.

