# Supplementary material for: Thoracic and vertebral deformities in lung transplantation: perioperative complications and long-term prognoses
Source: BMC Pulm Med. 2024 Jul 18;24:347. doi: 10.1186/s12890-024-03168-6 (PMC11256625; doi:10.1186/s12890-024-03168-6)
Supplement: Supplementary file 1 — Supplementary Material 1 [file 12890_2024_3168_MOESM1_ESM.pdf]

Supplemental table

Thoracic and Vertebral Deformities in Lung Transplantation: Perioperative Complications and Long-Term Prognoses

Risk Factors Associated with Volume Reduction

Variables	Odds Ratio	95%CI	P-value
Female	0.59	0.20–1.70	0.323
Age at LTx	0.97	0.93–1.02	0.193
Fibrosis	0.70	0.19–2.28	0.562
Thoracic deformity	3.91	1.22–12.65	0.021

CI, 95% confidence interval

Sensitivity analysis was conducted to assess the validity of the logistic regression models shown in Table 3, as selecting only two variables may fail to sufficiently account for relevant confounders.
